# A dataset of soil microstructure features and the weather conditions affecting them from 2005 to 2021 in the Caspian Depression

**DOI:** 10.1016/j.dib.2022.107957

**Published:** 2022-02-15

**Authors:** T. Romanis, M. Lebedeva, A. Kolesnikov, M. Sapanov, M. Sizemskaya

**Affiliations:** a Melnikov Permafrost Institute, 36 Merzlotnaya St, Yakutsk 677010, Russian Federation; b FRC V.V. Dokuchaev Soil Science Institute, 7 Pyzhevsky Lane, Moscow 109017, Russian Federation; c Institute of Forest Science RAS, 21 Sovetskaya St, Uspenskoe, Moscow Region 143030, Russian Federation

**Keywords:** Micromorphology, Soil complex, Air temperature, Wind, Rainfall, Relative humidity, Caspian Depression, Lowland, PPL, plane-polarized light, XPL, cross-polarized light

## Abstract

The soil cover of semi-desert territories is sensitive to modern climatic changes, responding to a change in the composition of soil complexes. Soil microstructure features reflect minor changes in microrelief or fluctuations in the level of groundwater. This property of soil microstructure to memorize soil formation conditions is used for subsequent characterization of changes in long-term climatic trends. But in semi-desert climates, it can be used as an indicator of short-term weather series. This article presents data collected on the territory of the Caspian Depression in the Dzhanybek Research Station of the Institute of Forest Science RAS. The soil cover of the studied site of this station is represented by a semi-desert, two-component meadow-steppe Solonetzic soil complex that strictly follows the elements of the local microtopography. The height range between the studied pits is 8 cm. The dataset includes micromorphological photographs of the state of the soil complex in 2005. This year is an initial moment for modeling soil properties at the present day taking into account the changed weather parameters. The weather data was collected between February 2005 and June 2021. It includes daily, decadal and monthly data related to air temperature, relative air humidity, wind speed and precipitation, soil temperature and depth of freezing (thawing) of the soil, snow cover depth. The measurement methods did not change throughout the entire observation period, which makes it possible to use the data to correct forecasts of the response of drylands to modern climatic changes with the subsequent verification of models in the field at present.

## Specifications Table

**Table:** 

Subject	Environmental Science
Specific subject area	Soil Science, Soil Evolution, Climate change
Type of data	Table, Image
How the data were acquired	The thin section analysis was conducted using an Olympus BX51 polarizing microscope (Tokyo, Japan) with an Olympus DP26 digital camera (Tokyo, Japan) in plane-polarized light (PPL), cross-polarized light (XPL). Microphotographs of all thin sections were done by using the Thixomet image analysis software. The weather parameters (relative air humidity, air and soil temperature, rainfall, wind force, depth of freezing-thawing of soil and height of snow cover) were measured at meteorological stations located in Dzhanybek (West Kazakhstan region, Kazakhstan; geographic coordinates are 49°25′26” N, 46°50′21” E) and Elton (Pallasovsky District, Volgograd Region, Russia; geographic coordinates are 49°07′32" N, 46°51′0" E). Air temperature was measured using mercury thermometer TM (measuring range from -35 °C to +50 °С with an accuracy of 0.1 °С). The maximum and minimum temperature was measured using applied thermometers TM1.1 and TM2.3 with a range of -35 ... + 50 °С and -50 ... + 40 °С, respectively, with an accuracy of 0.25 °С. The temperature of the deep layers of the soil was measured using a Savinov thermometer with an accuracy of 0.1 °С. The relative air humidity was determined using an M-19 hygrometer with an accuracy of 1%. The wind speed was measured by the combined direction and speed sensor MPV 502.14512 (measurement range from 0 to 35 m/s, accuracy of 2%). The amount of precipitation was determined using the O-1 rain gauge with an accuracy of 0.1 mm.
Data format	Raw data in table and raw image of soil microstructure
Description of data collection	The soil cover of the Dzhanybek Research Station of the Institute of Forest Science RAS (49° 23′57″ N 46° 47′46″) is represented by a semi-desert Solonetzic complex where the soils quite strictly follow the elements of the microrelief and correlate well with plant communities. Soil samples (bulk and undisturbed soil sample) were collected from horizons and morphons in a trench 3 m long to a depth of 1 m (Fig. 1). The collected bulk samples were dried, sieved and used for physicochemical analyses [2], [3]. The undisturbed soil samples were used to prepare thin sections [4].
Data source location	The soil sample site is located on the Dzhanybek Research Station territory of the Institute of Forest Science RAS on the Volga-Ural interfluve at a distance of 30 km north of Lake Elton within the northwestern drains of the Dzhanybek semi-desert plain. The absolute height of Dzhanybek Station is 28 m above sea level in the northwestern and 25.5 m above sea level in the western parts. Trench GPS coordinates: 49°23’42” N, 46°48’11” E.
Data accessibility	Repository name: *Mendeley Data* Data identification number: 10.17632/cfjrzm8zcb.1 Direct URL to data: https://data.mendeley.com/datasets/cfjrzm8zcb/1

## Value of the Data

• This dataset is the first to record the initial micromorphological properties of Solonetz complex soils and to indicate changes in the climatic characteristics of the arid territory (Caspian Depression) during the period from soil description in 2005 to the middle of 2021.
• This data is of interest to scientists studying the micromorphological features of the soil cover formed under current climate change conditions in the arid region.
• These data are useful for creating models of microstructural changes of arid soil correlating to climatic properties. It's a unique dataset for this because the researcher can verify the modeling results on a real object.

## 1 Data Description

**Fig. 1:**
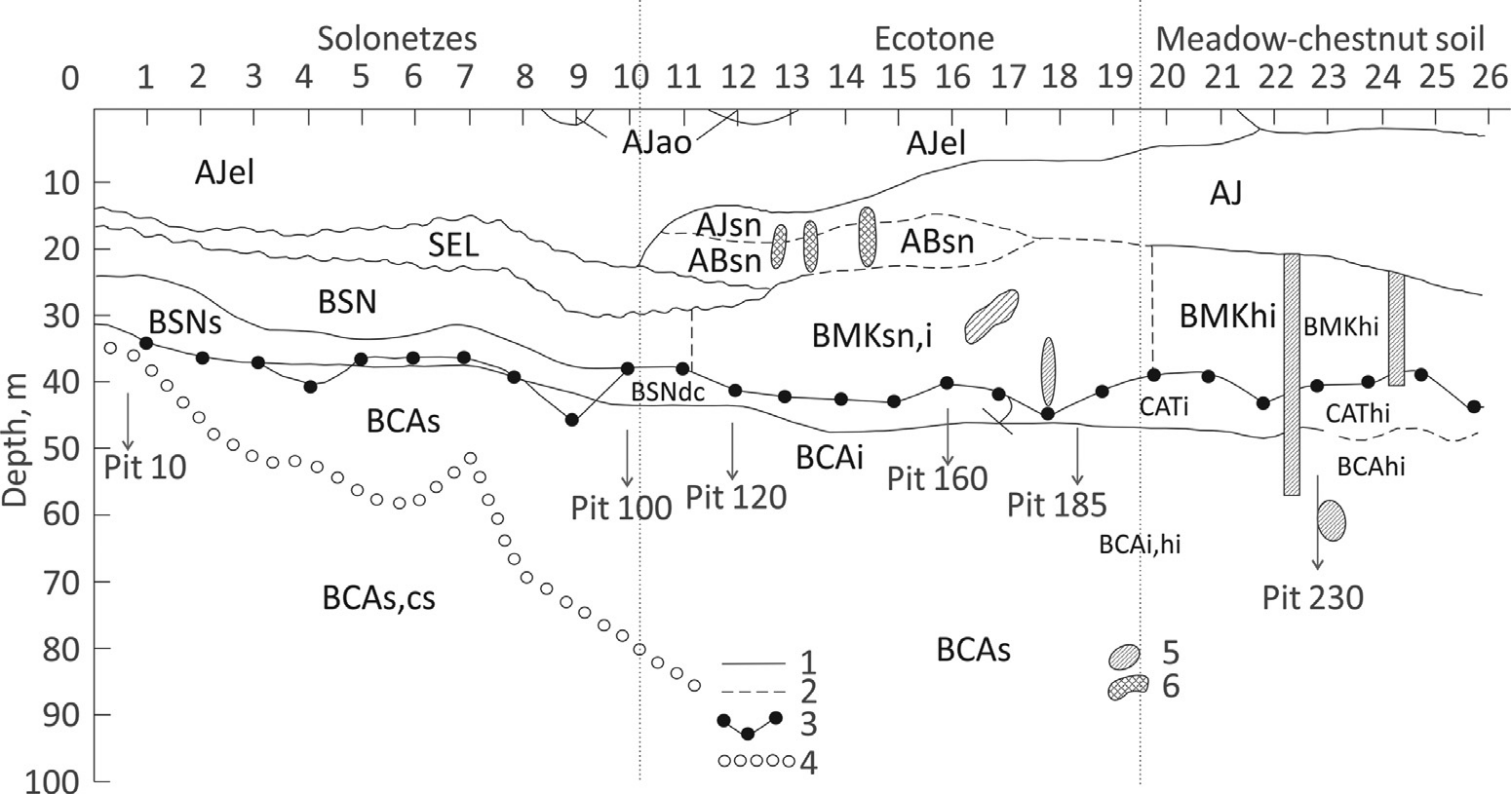
Distribution of soil horizons in the trench: 1 - abrupt boundary, 2 - diffuse boundary, 3 - line of effervescence, 4 - upper boundary of salt pedofeatures, 5 - dark-colored krotovinas, 6 - brown-colored krotovinas. Genetic horizons: AJ - Light humus horizon, SEL - Solonetz-eluvial horizon, BSN - Solonetzic horizon, BMK - Xerometamorphic horizon, BCA - Accumulative-carbonate horizon. Genetic features: ao – coarse humus; el – eluvial; sn - solonetzic s – saline; cs- accumulation of secondary gypsum; dc – carbonates derived from the soil solution and precipitated in the soil (micrit carbonates); hi – illuvial humus; I – illuvial clay.

Fig. 1 presents the 3 m-long trench at Dzhanybek Research Station of the Institute of forest Science RAS (49°23′42″ N, 46°48′11″ E) with markers of the locations of the horizons and morphons of the soil complex. The figure shows the boundaries of horizons and morphons. The composition of soil complexes and the number of their components vary significantly from Solonetzes to Meadow-chestnut soil.

**Fig. 2:**
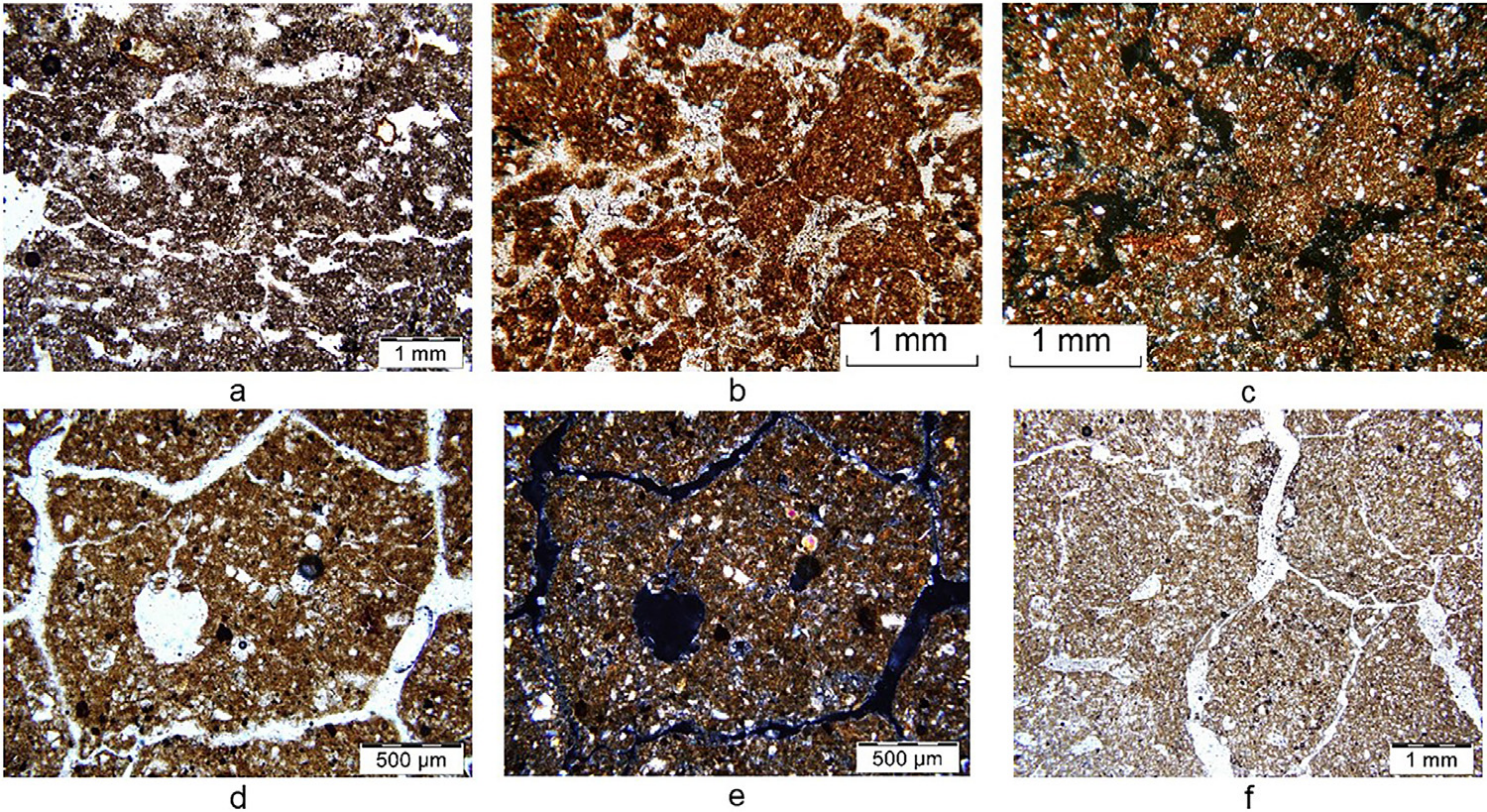
Microstructure of soil horizon of the ecotone: a – pit160_5-7 cm-AJ-PPL (3); b – pit160_21-23_cm-ABsn-PPL; c - pit160_21-23 cm-ABsn-XPL; d – pit160_27-29 cm-BMKsn,i-PPL (2); e – pit160_27-29 cm-BMKsn,i-XPL (2); f – рit160_43-48 cm-BSNdc-PPL.

The dataset consists of 40 soil microstructure images in plane-polarized light (PPL) and 35 soil microstructure images in cross-polarized light (XPL). All images are sorted into 6 folders (from 10 to 230 pit. The folders title means the number of soil pits in the trench (the location in the trench is shown in Fig. 1). Solonetzic soil complex in the trench includes Pit 10 medium-deep solonetz, Pit 100 deep solonetz, Pit 120 soil of the ecotone zone, Pit 160 soil of the ecotone zone, Pit 185 soil of the ecotone zone, Pit 230, meadow-chestnut soil. Fig. 2 shows a small part of PPL and XPL images from the dataset. The images are marked according to the following general principle: the pit number comes first, – then the sampling depth, – then the soil horizon, and finally the type of light (plane-polarized light (PPL), cross-polarized light (XPL). The photo marking may contain numbers (2) or (3) if more than one pair of photos was required to describe the horizon. For example:

1. pit160_21-23 cm-ABsn-XPL: «This file is from horizon ABsn sampling depth 21-23 сm from soil pit 160 in the trench. The microstructures photo was made in cross-polarized lights. »
2. pit160_27-29_cm-BMKsn,i-PPL (2): «This file is from horizon BMKsn,i sampling 27-29 cm сm from soil pit 160 in the trench. The microstructures photo was made in plane-polarized light. (2) – this is the second photo from this depth and horizon in the folder.»

The micromorphology photos of the soil complex in the trench maked with consideration of changing microtopography, lithological heterogeneity, change of plant communities (Fig. 1). This is a set of images reflecting the variability of soil microstructure features in the trench as of the 2005 year at Dzhanybek Research Station. Characteristics of the shape and size of nodules, cutans, pore space, micromasses, and ets. can be traced under the most representative conditions for a given area. The photographs were not segmented so that the data set could be used for the valuation of soil microstructure features quantitative indicators in any software.

High-resolution climate predictions from the HadGEM3A global climate model of the atmosphere from 1975 to the end of the 21st century indicate a 7% increase in drylands of total land area by 2100 while maintaining current dynamics of climate variability [1]. With the current trend of increasing the degree of aridization, the soil cover is changing. And under these conditions, this data-set characterizes the microstructure of soils, the closest to the beginning of the increase in the degree of climate aridity located at the Dzhanybek Station. Any modern soil pit or trench will not fully correspond to that described in 2005.

Thus data set is the "initial stop-moment" for analyzing the change in the features of the microstructure of the arid soil complex over time will allow researchers to quantify changes in the microstructure of soils in the feature.

If the predictive models turn out to be correct, then the trend of aridization will last until the end of the 21st century, which will make it possible to track the depth of transformation of the signs of microstructure and the degree of soil destruction over time and to assess the resistance of soils to climate change in the future with using this data-set. The chemical and physical indicators of soil pits data are given in Table 1 as of the 2005 year.

**Table 1:** Chemical properties of the soil complex in the trench.

				Corg	СО_3_equiv.	HCO_3_^−^	Cl^−^	SO_4_^2−^	Ca^2+^	Mg^2^	Na^+^	K^+^	
						
						
Pit	Horizon	Samples depth, cm	pH_H2O_	%		meq/100 g of soil							Sum ofsalts, %
10	AJel	0-6	7,7	2,69	-	0,25	0,01	0,08	0,03	0,3	0,22	0,07	0,03
	SEL	13-16	8,1	0,0	-	0,23	0,05	0,08	0,01	0,14	0,37	0,03	0,03
	BSN	21-26	8,8	0,81	-	0,62	0,48	0,46	0,19	0,01	1,60	0,1	0,12
	BSNs,s,	35-40	9,2	1,33	1,27	0,79	0,98	4,64	0,59	0,51	6,74	0,01	0,53
	BCAs, sn	50-56	9,1	-	1,8	0,45	1,00	16,2	5,65	4,15	8,69	0,05	1,21

100	AJel	0-10	7,4	2,9	-	0,23	0,01	0,06	0,29	0,11	0,09	0,04	0,03
	AJel	17-20	7,5	2,48	-	0,2	0,01	0,04	0,1	0,23	0,16	0,02	0,02
	SEL	26-30	7,8	2,54	-	0,23	0,01	0,01	0,1	0,01	0,35	0,03	0,03
	BSN	33-40	8,7	2,33	0,2	0,3	0,01	0,04	0,04	0,14	0,43	0,04	0,04
	BCAs	50-56	9,3	-	2,06	1,4	0,2	0,02	0,15	0,35	0,87	0,03	0,12
	BCAs,cs	82-87	9,1	-	2,68	0,45	1,62	11,52	1,41	2,54	9,24	0,03	0,91

120	AJel	0-10	7,7	3,17	-	0,43	0,04	0,02	0,35	0,27	0,08	0,07	0,04
	AJsn	17-20	8,2	2,12	-	0,53	0,09	0,02	0,2	0,35	0,16	0,02	0,05
	BMKsn	23-40	8,1	0,87	0,16	0,41	0,07	0,02	0,15	0,32	0,17	0,03	0,04
	CAThi	40-50	8,4	0,1	0,2	0,45	0,05	0,02	0,15	0,25	0,22	0,04	0,04
	BCA	50-55	9,1	0,04	1,77	0,91	0,02	0,02	0,15	0,5	0,63	0,03	0,08

160	AJel	0-13	7,7	2,75	-	0,39	0,04	0,02	0,3	0,45	0,08	0,09	0,04
	AJBsn	17-25	7,6	0,66	-	0,38	0,05	0,02	0,25	0,35	0,05	0,05	0,04
	BMKsn,i	27-42	7,9	1,08	0,08	0,37	0,05	0,02	0,1	0,4	0,09	0,03	0,04
	CAT i	43-48	8,5	0,24	1,32	0,67	0,05	0,02	0,5	0,55	0,17	0,04	0,07
	BCAi	50-55	8,6	0,63	2,3	0,71	0,07	0,02	0,4	0,5	0,24	0,04	0,07
	BCAs	82-87	9,1	0,0	2,93	1,03	0,06	0,02	0,15	0,35	0,66	0,02	0,09

185	AJel	0-10	8,2	4,43	-	0,27	0,06	0,04	0,15	0,35	0,05	0,1	0,03
	AJ	13-19	8,3	2,96	-	0,29	0,05	0,04	0,2	0,45	0,04	0,05	0,03
	BMKsn,i	27-40	8,2	1,6	-	0,3	0,04	0,04	0,25	0,35	0,05	0,04	0,03
	CAThi	43-48	8,0	0,14	2,06	0,52	0,04	0,04	0,25	0,3	0,13	0,04	0,05
	BCAi,hi	50-55	0,0	0,0	2,23	0,67	0,05	0,04	0,35	0,3	0,1	0,04	0,06

230	AJao	5-10	7,2	4,53	-	0,3	0,01	0,06	0,13	0,35	0,03	0,12	0,03
	AJ	13-19	8,0	2,44	-	0,53	0,01	0,06	0,49	0,34	0,03	0,11	0,05
	BMKhi	27-32	7,9	2,33	-	0,25	0,01	0,04	0,23	0,27	0,03	0,06	0,03
	CAThi	42-47	8,3	1,2	1,32	0,85	0,12	0,02	0,45	0,45	0,09	0,07	0,08
	BCAhi	50-55	8,4	1,38	1,73	0,84	0,08	0,02	0,4	0,55	0,09	0,06	0,07

**Fig. 3:**
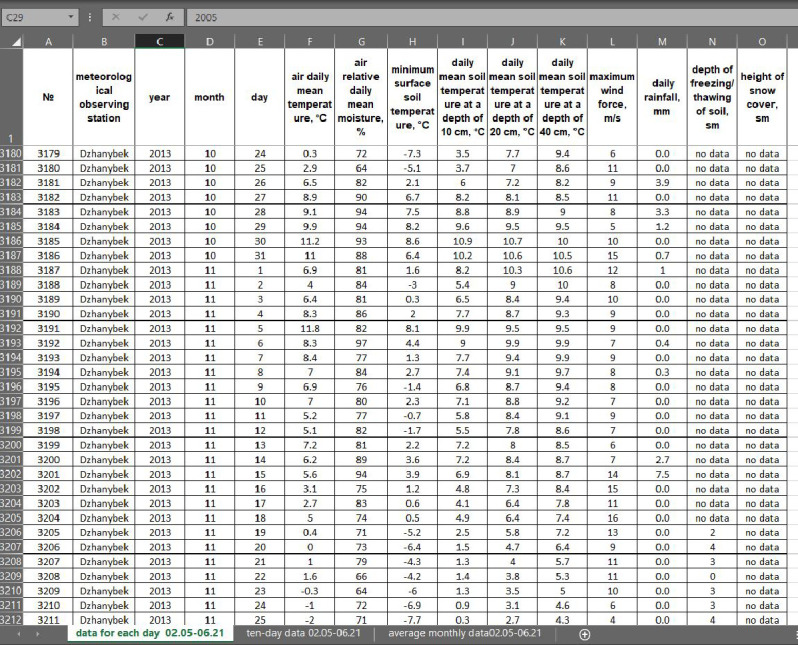
Distribution of the observation parameters in the Excel file across three Excel sheets: 1 – daily mean data; 2 – ten-day data; 3 – average monthly data, «no data» means «the researchers did not take measurements during this period».

The dataset includes the weather parameters which have influenced this soil complex during the last 16 years (relative air humidity, air and soil temperature, rainfall, wind force, depth of freezing-thawing of soil, height of snow cover). Fig. 3 presents the type of organization the weather conditions in file.

## 2 Experimental Design, Materials and Methods

The soils of the Dzhanybek Research Station are formed on a Late Pleistocene marine plain covered by a layer of brown loess-like calcareous loams which act as soil parent material. The representative site was selected based on microrelief analysis: the trench crosses the elements of the local microtopography with the amplitude of the heights being about 8 cm. This height difference caused differentiation of the two-component Solonetzic soil complex in the trench [5]. The length of the trench is 3 m, the width is 1 m. The vegetation consists of virgin steppe communities: the fescue–prostrate cypress association is confined to the micro elevations, fescue grass with inclusions of crested wheat grass (*Agropyron cristatum*) occupies the ecotone zone, and various forbs with crested wheat grass cover the microdepression. The Classification and Diagnostics of Russian Soils [5] was used for the diagnosis of horizons and morphons in the field. For each element we took measurements of its depth and thickness. The diagnosed horizons of the soil profiles which reflect the first reactions to changes in climatic conditions were: Humus (AJ) horizons, Eluvial horizon (SEL), the Solonetzic horizon (BSN), and the Subsolonetzic calcareous horizon (Xerometamorphic – BMK and Accumulative-carbonate – BCA) horizons (Fig. 1) [6]. Samples from the soil were collected from six vertical columns (pits) from every horizon in triplicate (Fig. 1).

The soil chemical analyses were performed in the Analytical Laboratory of the Dokuchaev Soil Science Institute according to standard methods [2,3]. The air-dried bulk samples were finely crushed after first removing the roots. Soil pH values were analyzed using the potentiometric method with a soil-to-water ratio of 1:2.5, after which the content of ions in the aqueous extract was measured. Total organic carbon (Corg) was determined using wet oxidation with potassium dichromate and concentrated sulfuric acid according to Turin's method, which is very close to the Walkley-Black method and is based on the wet oxidation of organic substance in a mixture of 0.4 N K_2_Cr_2_O_7_ and concentrated H_2_SO_4_ (1:1) at 150 °C for 20 min. The measurements were performed by photometry on a SPECOL 211 spectrometer at 590 nm.

CaCO3 equivalent was calculated on a base of volumetric determination of CO_2_ of the carbonates in modification by Kozlovskii. A soil sample was treated with 2 M HCl; the released CO_2_ was absorbed by a 0.4 M NaOH solution. Then, a saturated BaCl_2_ solution was added to the tube with NaOH, and the excess of alkali was titrated with 0.2 M HCl [2]. The obtained values of the carbonate ion concentrations were recalculated for calcium carbonates.

The dataset objects in the studied soil complex consists of Solonetzes (SN) within the elevated part, a meadow-chestnut soil (MCh) from the microdepression and an ecotone zone between them. At the beginning of the trench (pit 10), a medium-deep solonchakous Solonetz is found, which is gradually replaced by a deep Solonetz (pit 100). In the ecotone zone soils were strongly disturbed by earth burrowers. Data on soil properties of soil complex published earlier [7] has been included for comparison.

The undisturbed soil samples were impregnated with epoxy resin (the refractive index of 1.536) for micromorphological analysis preparation. After that the hardened monoliths were cut into small blocks and reduced down to about 30 μm [4]. The thin section analysis was conducted using an Olympus BX51 polarizing microscope (Tokyo, Japan) with an Olympus DP26 digital camera (Tokyo, Japan) in plane-polarized light (PPL), cross-polarized light (XPL). Microphotographs of all thin sections were acquired by using the Thixomet image analysis software.

Since the description and analysis of the soil complex, data on climatic parameters have been collected for 16 years. The soils of the Solonetz complex are sensitive to the slightest changes in environmental parameters, with which the degree of aridity increases or the groundwater rises to the surface. For model experiments on changes in the structure and properties of the soil complex in a changing climate, weather parameters were collected for the last 16 years. The weather parameters (relative air humidity, air and soil temperature, rainfall, wind force, depth of freezing-thawing of soil and height of snow cover) were measured at meteorological stations located in Dzhanybek (49° 25′26″ N, 46°50′21″ E) and Elton (Pallasovsky District, Volgograd Region, Russia; geographic coordinates are 49°7′32″ N, 46°51′0″ E). Air temperature was measured using mercury thermometer TM (measuring range from -35 °C to +50 °С with an accuracy of 0.1 °С). The maximum and minimum temperature was measured using applied thermometers TM1.1 and TM2.3 with a range of -35 ... + 50 °С and -50 ... + 40 °С, respectively, with an accuracy of 0.25 °С. The temperature of the deep layers of the soil was measured using a Savinov thermometer with an accuracy of 0.1 °С. The relative air humidity was determined using an M-19 hygrometer with an accuracy of 1%. The wind speed was measured by the combined direction and speed sensor MPV 502.14512 (measurement range from 0 to 35 m/s, accuracy of 2%). The amount of precipitation was determined using the O-1 rain gauge with an accuracy of 0.1 mm.

## Ethics statements

The authors declare that they have no known competing financial interests or personal relationships which have or could be perceived to have influenced the work reported in this article.

## CRediT authorship contribution statement

**T. Romanis:** Writing – original draft, Visualization. **M. Lebedeva:** Investigation, Writing – original draft. **A. Kolesnikov:** Data curation. **M. Sapanov:** Investigation. **M. Sizemskaya:** Validation, Resources.

## Declaration of Competing Interest

The authors declare that they have no known competing financial interests or personal relationships that could have appeared to influence the work reported in this paper.
